# Organized Structure of Ventricular Fibrillation during Prolonged Heart Perfusion in Dogs

**DOI:** 10.17691/stm2020.12.3.03

**Published:** 2020-06-28

**Authors:** M.I. Guryanov, R.S. Pusev, N.M. Guryanova, E.A. Kharitonova, P.K. Yablonsky

**Affiliations:** Professor, Department of Basic and Specific Medical Sciences, Faculty of Medicine; Saint Petersburg State University, 7**–**9 Universitetskaya Naberezhnaya, Saint Petersburg, 199034, Russia; Associate Professor, Department of Informatics, Saint Petersburg School of Physics, Mathematics, and Computer Science; National Research University Higher School of Economics, 16 Soyuza Pechatnikov St., Saint Petersburg, 199008, Russia; PhD Student, Department of Pharmacology, Faculty of Medicine; Saint Petersburg State University, 7**–**9 Universitetskaya Naberezhnaya, Saint Petersburg, 199034, Russia; Associate Professor, Department of Basic and Specific Medical Sciences, Faculty of Medicine; Saint Petersburg State University, 7**–**9 Universitetskaya Naberezhnaya, Saint Petersburg, 199034, Russia; Professor, Director; Saint Petersburg Research Institute of Phthisiopulmonology, Ministry of Health the Russian Federation, 2**–**4 Ligovsky Avenue, Saint Petersburg, 191036, Russia; Head of the Department of Hospital Surgery; Saint Petersburg State University, 7–9 Universitetskaya Naberezhnaya, Saint Petersburg, 199034, Russia; Dean of the Faculty of Medicine; Saint Petersburg State University, 7**–**9 Universitetskaya Naberezhnaya, Saint Petersburg, 199034, Russia

**Keywords:** ventricular fibrillation, organized structure of ventricular fibrillation, perfusion.

## Abstract

**The aim of the study** was to identify the organized ventricular fibrillation (VF) activity in the dog heart and characterize its quantitative parameters during prolonged heart perfusion.

**Materials and Methods.:**

A total of four experiments on isolated dog’s hearts perfused with the blood of a supporting dog were performed. Episodes of VF were recorded in the form of an electrogram followed by a spectral analysis by the fast Fourier transform in the range of 0.5–15 Hz. The frequency, spectral power (amplitude), and relative weight (%) of the 1^st^, 2^nd^, and 3^rd^ highest amplitude oscillations were determined (frequency — mode; amplitude, relative weight — M±SEM; n=120).

**Results.:**

In the perfused dog heart, VF was characterized by an organized activity, as evidenced by the dominant structure of the oscillation frequencies. Oscillations with a frequency of 9–10 Hz (occurring in 1/10 of the 0.5–15 Hz range) represent 42–44% of the spectral power and dominate the structure of the oscillation frequencies. The frequency and spectral power of the dominating oscillations proved to be stable thus indicating that under perfusion, VF did not cause disturbances in the heart organized activity.

**Conclusion.:**

Our experimentation with isolated perfused hearts revealed the patterns of VF that could not be revealed *in situ* under conditions complicated by nerve factors and ischemia in VF. The results of the work are protected with a patent which is applicable for VF diagnosis in implantable defibrillators.

## Introduction

Unlike coordinated heart contractions that support hemodynamics, ventricular fibrillation (VF) is characterized by uncoordinated contractions of individual myocardial fibers which unable to maintain hemodynamics and lead to death [1, 2]. VF-associated sudden heart death contributes to 15–20% of deaths worldwide [3–5]. Therefore, VF continues to be an urgent medical problem.

Although ventricular fibrillation is traditionally considered to be a disorganized process [6–9], organized activity in VF have been recently found [10–12]. This activity decreases during the first 8–10 min of VF, and then VF becomes disorganized [13–15]. It has been found that this decrease in the organized activity is associated with an inhibition of the myocardial electrical activity caused by VF-induced ischemia [10–15]. This inhibition would not occur during perfusion, which prevents heart ischemia. Indeed, as shown by optical mapping, the dominant VF frequency of 10–13 Hz remained stable during cardiac perfusion [16]. However, the optical mapping covered no more than 8 cm^2^ of the epicardium surface of the left ventricle; moreover, optical recording was performed for 5 s every 5 min of perfusion, which is no more than 1 min of recording during 1-hour perfusion. It remained unclear whether the above results were applicable to the entire ventricular myocardium and to the entire 1-hour perfusion period. Another study indicated that VF was stable during 1-hour perfusion [17], but this statement was not supported by quantitative data.

Thus, we were unable to find published reports on the organized VF activity and its quantitative parameters under heart perfusion. Such parameters though would be of great importance for automatic VF diagnosis in implantable defibrillators. These devices sometimes fail to produce a discharge under VF causing sudden death of a patient; in other cases, 20% of patients suffer from undue discharges provoked by a false VF diagnosis [18–21].

**The aim of the study** was to identify the organized ventricular fibrillation activity in the dog heart and characterize its quantitative parameters during prolonged heart perfusion.

## Materials and Methods

A total of four experiments in dogs were performed. Keeping animals and conducting experiments was carried out in accordance with the Guide for the Care and Use of Laboratory Animals (National Research Council, 2011), as well as with the ethical principles of the European Convention for the Protection of Vertebrate Animals used for Experimental and Other Scientific Purposes (Strasbourg, 2006). In dogs weighing 20–30 kg under thiopental anesthesia (10–15 mg/kg — initial dose and 4–7 mg/kg — hourly), artificial lung ventilation (ALV) was performed using an iVent 201 apparatus (General Electric Healthcare, Israel). After thoracotomy, the aortic brachiocephalic trunks, pulmonary artery, vena cava, pulmonary veins, and the unpaired vein were isolated, ligated, and cut off. Before removing the heart from the chest, 500 IU/kg of heparin was injected into the femoral vein. A drainage tube was inserted through the superior vena cava into the right atrium, and through the pulmonary vein into the left atrium.

Then, the aorta was cannulated and the coronary arteries were perfused for 6–8 min with Custodiol cardioplegic solution (Dr. F. Köhler Chemie GmbH, Germany) at a temperature of 5°C. Following cardioplegia, the heart was perfused with the blood of a supporting dog (30–40 kg) that was anesthetized and mechanically ventilated. The time interval from the end of cardioplegia to the start of perfusion did not exceed 10 min as recommended [22].

**Figure 1:**
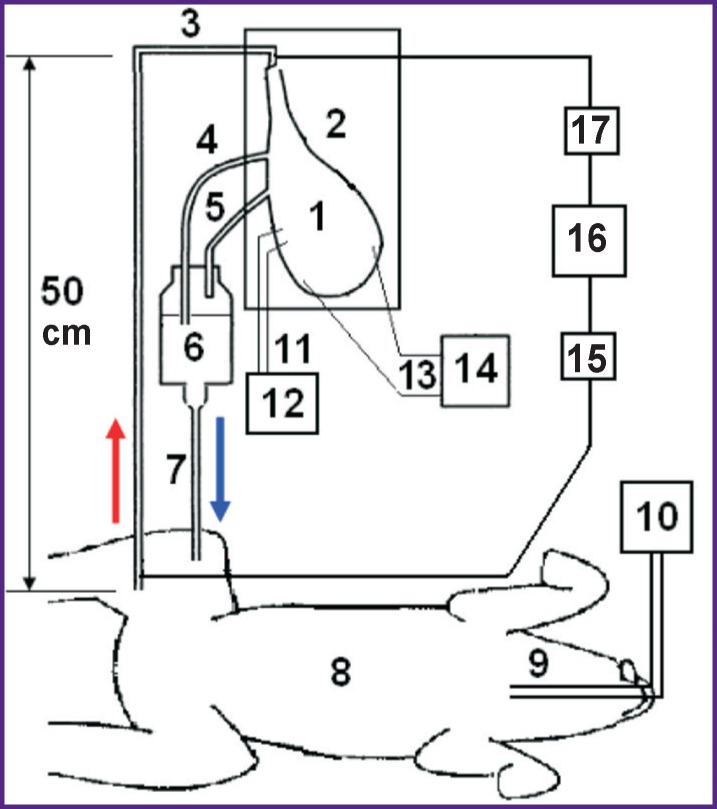
Scheme of the experiment with the isolated dog heart perfused with the blood of the supporting dog: *1* — heart; *2* — heart chamber; *3* — tube connecting aorta to femoral artery of the supporting dog; *4* and *5* — drainage tubes in the right and left atria; *6* — venous reservoir; *7* — drainage tube connecting venous reservoir to femoral vein of the supporting dog; *8* — supporting dog; *9* — endotracheal tube in trachea; *10* — ventilating machine; *11* — pacemaker electrodes; *12* — pacemaker; *13* — lead electrodes; *14* — recorder; *15* and *17* — pressure sensors in aorta and femoral artery; *16* — pressure monitor. The red arrow shows arterial blood flow through aorta of the isolated heart; the blue arrow indicates return of venous blood to femoral vein of the supporting dog

From the femoral artery of the supporting dog, the blood entered the aorta of the isolated heart (Figure 1). Aortic perfusion pressure was maintained (using a clamp) at 90–100 mm Hg, which led to the closure of the aortic valve; as a result, coronary perfusion was set up according to the Langendorff principle [23, 24]. The pressure was measured using Combitrans pressure transducers (Braun, Poland), connected to a Datex-Ohmeda Cardiocap/5 monitor (General Electric Healthcare, Finland). Venous blood from the right and left atria flowed down the drainage tubes into a reservoir installed above the supporting dog, and from the reservoir into the femoral vein of the supporting dog. To prevent thrombosis the supporting dog was heparinized (500 IU/kg — initial dose and 150 IU/kg — hourly).

The heart was placed into a transparent plastic chamber, which allowed for visual control. The temperature in the chamber was maintained at 37°C using a MN-2000 (China) temperature controller. The discrepancy between the temperature control readings and the PTS-10M reference thermometer (EtalonPribor, Russia) did not exceed 0.1°C in a range of 25–45°C. The supporting dog was heated with an electric blanket. The body temperature of the dog was maintained at 37°C using the MN-2000 temperature controller.

In all four dogs, the frequency-amplitude parameters of VF were determined under conditions of heart perfusion. Electric signals from electrodes inserted into the right and left ventricles were recorded using a Cardiotechnika-ECG-8 cardiograph (Inkart, Russia) at a sampling rate of 1000 Hz. There were no pathological changes in the ventricular electrograms before the start of VF. VF was induced by electric stimuli with a frequency of 10 Hz and an amplitude of 10 mA produced by a pacemaker (see Figure 1). We performed a frequency-amplitude analysis of 12-second segments of ventricular electrogram using the fast Fourier transform at 30 frequencies of 0.5 Hz width in the range 0.5–15 Hz: 0.5, 1, 1.5, ..., 15 Hz.

The frequency (Hz), spectral power (amplitude, mV), and relative weight (%) of the 1^st^, 2^nd^, and 3^rd^ highest amplitude oscillations were determined (frequency — mode; amplitude, relative weight — M±SEM). This spectral analysis technique has been developed in this lab [13–15] and now it is used for the first time.

**Statistical data processing** was performed in The R Project for Statistical Computing, version R 3.5.3 [25]. This program is included in the list of the best software developments [26]. Statistical processing was carried out by nonparametric methods using a comparison of the frequency-amplitude parameters of the VF according to the χ^2^ criterion and Spearman correlation [27]. Data are presented as arithmetic mean±standard error of the mean (M±SEM, n=120). Differences were considered statistically significant at p≤0.05.

## Results

**Figure 2:**
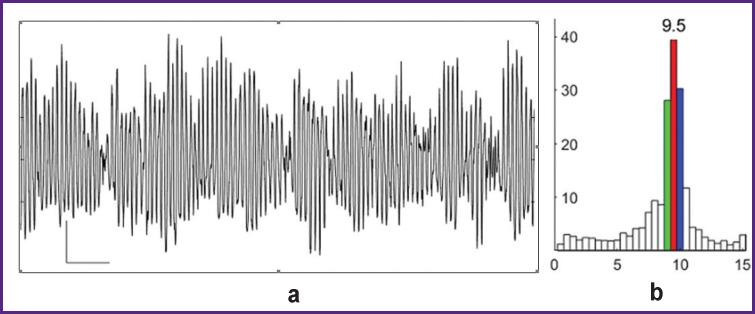
Electrogram (a) and spectrogram (b) of ventricular fibrillation during perfusion of the dog’s heart Calibration of the electrogram: 2 mV; 1 s. In the spectrogram: the abscissa — frequency (Hz); the ordinate — amplitude (mV)

In VF, the electrogram of the perfused heart is dominated by oscillations of 9–10 Hz (Figure 2 (a)); the spectrum of oscillation frequencies shows the 9.5, 10, and 9 Hz as generating the first, second and third highest spectral power and dominating the structure of the oscillation spectrogram (Figure 2 (b)).

**Figure 3:**
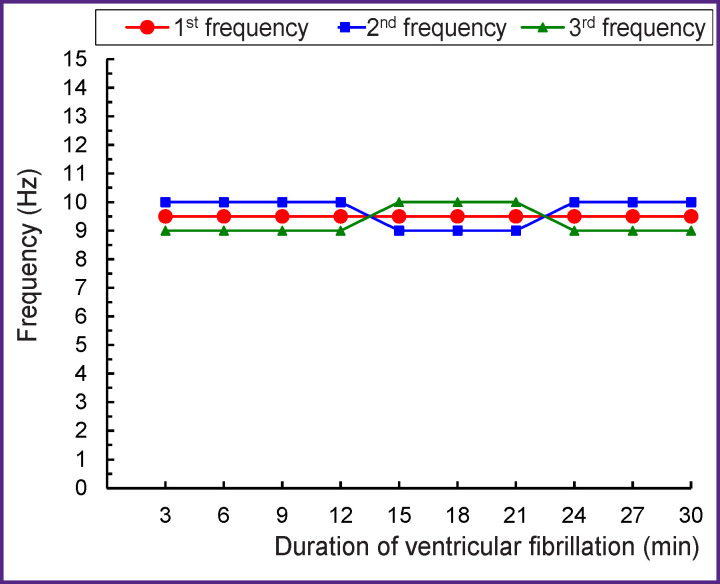
The frequencies of the 1^st^–3^rd^ highest amplitude oscillations in ventricular fibrillation during perfusion of the dog’s heart; mode, n=120

The dominant frequency structure detected in the 12-second interval of VF (see Figure 2) holds for the 30-minute interval of VF (Figure 3). The 1^st^ frequency (with the highest spectral power) has a constant value of 9.5 Hz, and the 2^nd^ and 3^rd^ frequencies are 0.5 Hz above or below the 1^st^ frequency (see Figure 3). With 30-minute perfusion of the fibrillating heart, these three frequencies form a continuous stable band of dominant frequencies of 9–10 Hz with a width of 1.5 Hz, i.e., 1/10 of the 0.5–15 Hz range.

**Figure 4:**
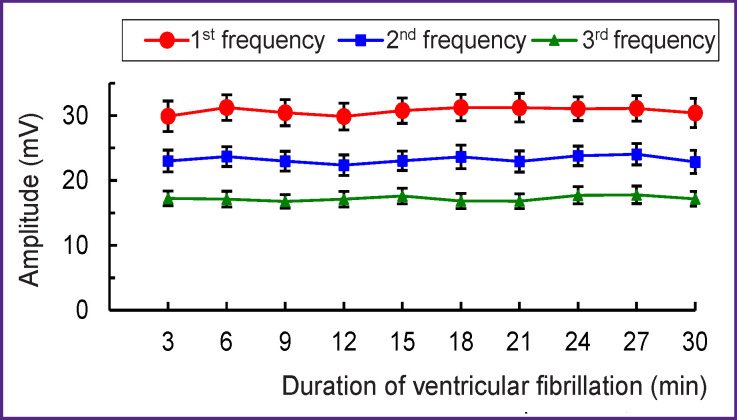
The amplitudes of the 1^st^–3^rd^ highest amplitude oscillations in ventricular fibrillation during perfusion of the dog’s heart M±SEM, n=120; p>0.05 when comparing pairs of values of the 1^st^–3^rd^ frequency

The amplitude of the 1^st^ frequency oscillations is 30–31 mV, that of the 2^nd^ frequency 23–24 mV, and for the 3^rd^ one — 17–18 mV. These amplitude values are practically stable — the minimal variations within the 3-minute intervals of VF are not significant (Figure 4).

**Figure 5:**
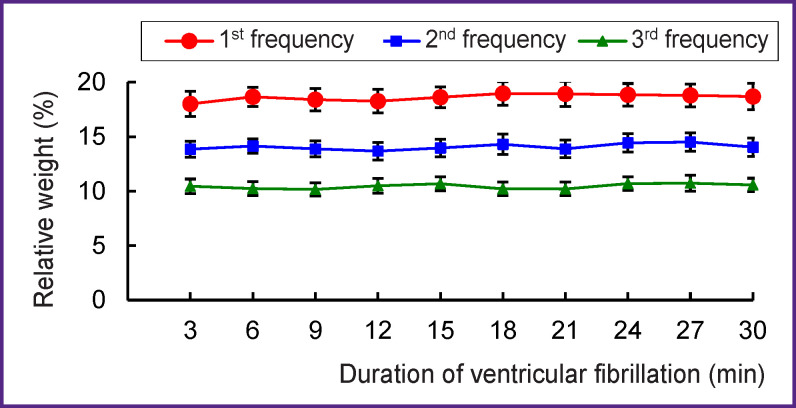
Relative weight of the 1^st^–3^rd^ highest amplitude oscillations in ventricular fibrillation during perfusion of the dog’s heart M±SEM, n=120; p>0.05 when comparing pairs of values of the 1^st^–3^rd^ frequency

Oscillations of the 1^st^ frequency, taking about 1/30 of the 0.5–15 Hz range, generate 18–19% of the total spectral power, for the 2^nd^ frequency, it is 13–14%, and for the 3^rd^ — 10–11%; the relative weight of the oscillations at each of these frequencies is practically stable: the changes within the 3-minute intervals of VF are not significant (Figure 5). The specific weight of the oscillations at each frequency correlates with the amplitude of the oscillations at these frequencies. There is a strong direct correlation between the amplitude and relative weight of oscillations within the 30-minute perfusion of the heart: at the 1^st^ frequency, it is 0.79; at the 2^nd^ — 0.83, and at the 3^rd^ — 0.94 (p<0.01).

In summary, the oscillations of the 1^st^–3^rd^ frequencies, taking together about 1/10 of the 0.5–15 Hz range, generate 42–44% of the total spectral power and dominate in the frequency structure. The dominant structure is stable in frequency, amplitude, and relative weight of the dominant oscillations of the 1^st^–3^rd^ frequencies.

## Discussion

Under perfusion, VF in the isolated dog heart is characterized by the dominant structure of oscillation frequencies (see Figure 2), which indicates an organized (synchronized) myocardial activity. If cardiomyocytes generated random action potentials independently from each other, these potentials, randomly summing up, would give a random total process with a uniformly distributed spectral density in the range of 0.5–15 Hz.

The dominance of VF oscillations with a frequency of 9–10 Hz reflects, apparently, the synchronized activity of cardiomyocytes generating action potentials with a frequency of 9–10 Hz. This hypothesis is supported by the observation that cardiomyocytes generated action potentials with a frequency of 9–10 Hz in the first seconds of the VF [11, 12]; in this first short period, the myocardium is not ischemic yet, thanks to resources of ATP and oxygen [22, 28].

The stability of the frequency and spectral power of the dominant oscillations (see Figures 3–5) indicates that VF does not disorganize the electrical activity in the perfused heart. The presented results could only be obtained with the isolated perfused heart model but not with the heart *in situ* where nerve factors and ischemia complicate the analysis of VF. The obtained results can be used for VF diagnosing, since the frequency of oscillations in the fibrillating dog’s heart is close to that in humans [11, 12, 29–31]. In addition, an electrogram reflects the global activity of the ventricles by analogy with the ECG [32]. The fast Fourier transform used in this study allows one to determine the frequency-amplitude structure of the VF electrogram; this structure was shown to be similar to that of the ECG recorded in VF [8, 10, 11]. The results of the work are protected with a patent [33], and they can be used for automatic VF diagnosis in implantable defibrillators.

## Conclusion

Ventricular fibrillation of the isolated dog heart under perfusion is characterized by an organized activity, as evidenced by the existence of a dominant structure of the oscillation frequencies. Oscillations with a frequency of 9–10 Hz, taking 1/10 of the 0.5–15 Hz range, generate 42–44% of the spectral power and dominate the frequency structure of the VF. The stability of the frequency and spectral power of the dominant oscillations indicates that, under perfusion, VF caused no disturbances in the organized myocardial activity.

The results of the work show that using the isolated artificially perfused heart makes it possible to identify patterns of VF that cannot be seen with the heart *in situ* where the analysis of VF is complicated by nerve factors and ischemia.
